# Supplementary material for: Alternative Promoter Use Governs the Expression of IgLON Cell Adhesion Molecules in Histogenetic Fields of the Embryonic Mouse Brain
Source: Int J Mol Sci. 2021 Jun 28;22(13):6955. doi: 10.3390/ijms22136955 (PMC8268470; doi:10.3390/ijms22136955)
Supplement: Supplementary file 1 [file ijms-22-06955-s001.zip › ijms-1236210-supplementary.pdf]

Supplementary Material

Alternative Promoter Use Governs the Expression of IgLON Cell Adhesion Molecules in Histogenetic Fields of the Embryonic Mouse Brain

Toomas Jagomäe[1,2,3,†], Katyayani Singh[12, ,†,*] Mari-Anne Philips[1,2], Mohan Jayaram[1,2], Kadri Seppa[1,2,3], Triin Tekko[4], Scott F. Gilbert[5], EeroVasar[1,2] and Kersti Lilleväli[1,2]

[1] Department of Physiology, Institute of Biomedicine and Translational Medicine, University of Tartu, 19 Ravila Street, 50411 Tartu, Estonia;toomas.jagomae@gmail.com (T.J.); marianne.philips@ut.ee (M.-A.P.);jmohan80@gmail.com (M.J.); kadriseppa@gmail.com (K.Se.); eero.vasar@ut.ee (E.V.); kersti.lillevali@ut.ee (K.L.)

[2] Centre of Excellence in Genomics and Translational Medicine, University of Tartu, Tartu, Estonia

[3] Laboratory Animal Centre , Institute of Biomedicine and Translational Medicine, , University of Tartu, 14B Ravila Street, 50411 Tartu, Estonia[4] The Instituto Gulbenkian de Ciência, Rua da Quinta Grande 6, 2780-156 Oeiras, Portugal; ttekko@igc.gulbenkian.pt

[5] Department of Biology, Swarthmore College, Swarthmore, PA, 19081, USA; sgilber1@swarthmore.edu

* Correspondence: katyayani.micro@gmail.com; Tel.: +372-7374-335

† These authors have contributed equally to this work.

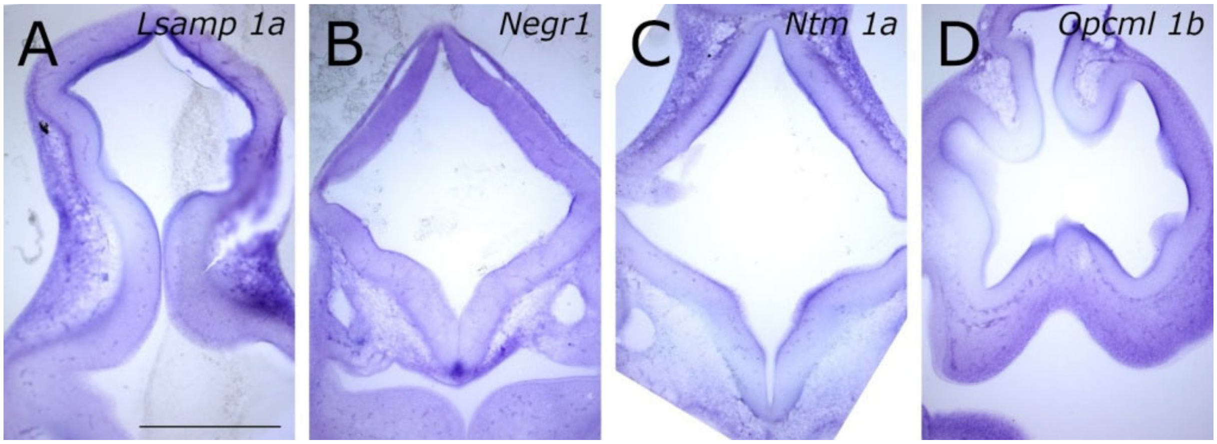

Supplementary Figure S1. In situ mRNA hybridization displaying *Lsamp 1a*, *Negr1*, *Ntm 1a* and *Opcml 1b* at E10.5 coronal sections. (A) signal from *Lsamp 1a* probe is intense in the ventricular zone. (B) Intense expression of *Negr1* is observable at the floor plate. (C) *Ntm 1a* expression is established dorsally in the ventricular zone and in the developing ganglionic eminences. (D) singal from *Opcml 1b* probe is observable throughout the ventricular zone of the developing nervous system. Scale bar: 1 mm.

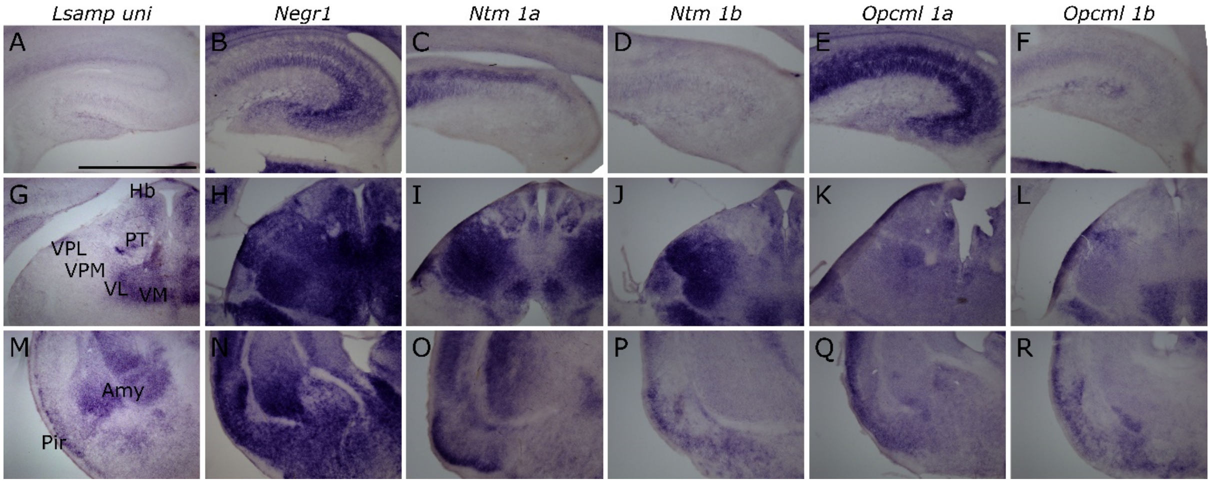

Supplementary Figure S2. Expression from IgLON family alternative promoters at E17 forebrain. Expression of *Lsamp uni*, *Negr1*, *Ntm 1a*, *Ntm 1b*, *Opcml 1a*, and *Opcml 1b* mRNA was detected by in situ mRNA hybridization on coronal cryo-sections. Expression of corresponding probes at the level of **(A – F)** hippocampus, **(G – L)** dorsal thalamus, and **(M – R)** piriform cortex. Signal of **(A)** *Lsamp uni*, **(D)** *Ntm 1b*, and **(F)** *Opcml 1b* is similarly faint throughout the hippocampus formation. Signal from (B) *Negr1* and **(C)** *Ntm 1a* form continuous pattern where *Negr1* expression is stronger toward developing dentate gyrus and *Ntm 1a* toward cingulate cortex. Expression of **(E)** *Opcml 1a* is observable through the hippocampal formation. In developing thalamus **(G)** *Lsamp uni* probe is expressed nuclei surrounding the third ventricle (i.e Hb) including VM. This expression pattern is opposed by **(I)** *Ntm 1a* and **(J)** *Ntm 1b* where the strong expression is also observable in VPM and VM. Intense staining of **(H)** *Negr1* is observable in most of the thalamic nuclei with the exception of PT and VL which displays moderate staining. Expression of **(K)** *Opcml 1a* is rather uniform throughout the nuclei whereas **(L)** *Opcml 1b* is observable in DL, VPL and VM being faint or absent

in other nuclei. **(M – R)** all transcripts are present in the piriform cortex being strongest in the second layer and generally dilutes towards deeper layers. Particularly the expression is absent in the bed nucleus of the external capsule. Abbreviations: Amy, amygdala; Hb, habenula; Pir, piriform cortex; PT, posterior thalamus: VM, ventromedial nucleus; VL, ventrolateral nucleus; VPL, ventral posterolateral nucleus; VPM, ventral posterior medial nucleus. Scale bar: 1 mm.
